# Survey Design to Monitor Drug Efficacy for the Control of Soil-Transmitted Helminthiasis and Schistosomiasis

**DOI:** 10.1093/cid/ciab196

**Published:** 2021-06-14

**Authors:** Luc E Coffeng, Bruno Levecke, Jan Hattendorf, Martin Walker, Matthew J Denwood

**Affiliations:** 1 Department of Public Health, Erasmus MC, University Medical Center Rotterdam, Rotterdam, The Netherlands; 2 Department of Virology, Parasitology and Immunology, Ghent University, Merelbeke, Belgium; 3 Swiss Tropical and Public Health Institute, Basel, Switzerland; 4 University of Basel, Basel, Switzerland; 5 Department of Pathobiology and Population Sciences and London Centre for Neglected Tropical Disease Research, Royal Veterinary College , Hatfield, United Kingdom; 6 Department of Veterinary and Animal Sciences, University of Copenhagen, Denmark

**Keywords:** soil-transmitted helminthiasis, schistosomiasis, drug efficacy, survey design, health economics

## Abstract

**Background:**

Control of soil-transmitted helminthiasis and schistosomiasis relies heavily on regular preventive chemotherapy. Monitoring drug efficacy is crucial to provide early warning of treatment failures. The World Health Organization (WHO) recommends a survey design in which only egg-positive individuals are retested after treatment. Although this practice makes more efficient use of resources, it may lead to biased drug efficacy estimates.

**Methods:**

We performed a simulation study to assess the potential for bias when evaluating drug efficacy using the World Health Organization–recommended survey design, and to identify alternative designs for evaluating drug efficacy that are less affected by bias. These designs were also based on selection of egg-positive individuals, but involve retesting them a second time at baseline and up to 2 times at follow-up. The utility of the different designs was compared fairly by constraining them to the same budget.

**Results:**

The standard procedure of selecting egg-positive individuals can introduce a substantial positive bias in drug efficacy due to regression toward the mean, particularly when infection levels or drug efficacy are low. This bias was completely eliminated by using a second baseline sample, conditionally on the first sample being excluded from analysis. Precision of estimates can be improved by increasing the number of thick smears and/or samples per person at follow-up, despite fewer individuals being tested within the same budget.

**Conclusions:**

We present optimized survey designs to monitor drug efficacy in field settings, which are highly relevant for sustained control of soil-transmitted helminths and schistosomiasis, as well as onchocerciasis and lymphatic filariasis.

Control of soil-transmitted helminths (STH; *Ascaris lumbricoides*, *Trichuris trichiura*, and hookworms *Ancylostoma duodenale* and *Necator americanus*) and schistosomes (SCH; *Schistosoma mansoni*, *Schistosoma japonicum*, and *Schistosoma haematobium*) relies heavily on preventive chemotherapy with either a benzimidazole (albendazole or mebendazole for STH) or praziquantel (for SCH) [1]. Given that drug resistance due to historical overuse or misuse of deworming drugs is widespread in veterinary helminth infections [2, 3], it is feared that drug resistance will also develop eventually in STH and SCH [4]. However, we currently do not have a good understanding of when and where this is most likely to happen. Surveillance of drug efficacy is therefore crucial, especially in settings with longstanding or highly frequent deworming of target populations [5].

Evaluation of drug efficacy requires measurement of infection levels before and after treatment [6]. Infection levels are typically measured by counting eggs in a sample of stool (STH, *S. mansoni*, and *S. japonicum*) or urine (*S. haematobium*) using a standard egg-counting method (stool: Kato-Katz thick smear [KK]; urine: urine filtration). Egg counts are typically expressed in terms of eggs per gram of stool (EPG) or eggs per 10 mL of urine [7]. Drug efficacy is commonly expressed as a cure rate (CR) or egg reduction rate (ERR). The CR is defined as the proportion of egg-positive individuals who became egg-negative after treatment [8–10]. The ERR is the relative difference in either the arithmetic or geometric mean count in before and after treatment [4, 11, 12]. The use of CR is commonly preferred in randomized clinical trials because of its lower sensitivity to outliers than the ERR and the fact that no distributional assumptions are needed. However, in field settings, the ERR is preferred because it is not as strongly affected by pretreatment infection levels as the CR (which is lower at higher pretreatment egg counts), making it more comparable across different settings or to established reference figures [11, 13]. In this study, we consider the group-based ERR, which is based on the relative difference in population mean counts before and after treatment, as this is the recommended way of calculating the ERR [4].

Typically, egg count measurements are performed longitudinally, meaning that the same individuals are tested before and after drug administration [11, 13–16]. To save resources, it is common practice to exclude individuals with zero pretreatment egg counts from further testing, both in drug trials and field settings [15, 16]. This is considered especially relevant in settings with low infection levels where the majority of baseline samples will turn out egg-negative, and the perception is that retesting these individuals (who are likely to retest negative) is a waste of resources. For example, in a setting with 10% prevalence, one might consider screening 1000 individuals (per trial arm) at baseline, select the 100 egg-positive individuals, and treat and retest these 100, leading to a total of 1100 tests. This is considered more cost-effective than testing 550 individuals twice (before and after drug administration), of whom only 55 would be positive at baseline.

The World Health Organization (WHO) currently recommends that estimates of drug efficacy in endemic populations targeted by preventive chemotherapy be based on surveys that select individuals who are egg-positive at baseline [6]. However, the practice of basing the efficacy estimate on the same counts that are used for selection should be expected to result in a type of statistical bias known as regression toward the mean [17]. This occurs due to a systemic overestimation of the pretreatment egg count resulting from a combination of random variation in egg counts within individuals over time (due to natural temporal variation in egg density and imperfect diagnostics) and the practice of excluding counts that do not meet a threshold value. This becomes obvious if considering a theoretical second set of samples taken on the same day from the included individuals: this second set of samples may well include zero counts and would therefore be expected to have a lower mean (on average) than the counts that were used to select these individuals. This mechanism inflates estimates of drug efficacy but is not considered to be a problem in randomized controlled trials as randomization ensures that any bias is equivalent across trial arms and therefore cancels out in the comparison of trial arms. However, in field surveys, this bias hinders comparison of drug efficacy estimates to a fixed reference value. Despite its potential importance, this bias has not previously been studied in field settings. We present results from bespoke simulation software to demonstrate and quantify the potential magnitude of this bias. We further propose alternative yet simple designs for surveillance of drug efficacy that aim to eliminate the source of bias while optimizing the use of limited resources.

## METHODS

### Overview

We designed bespoke simulation software to help study the statistical properties of a flexible spectrum of different study designs for anthelmintic drug efficacy surveys, and used this software to perform a simulation study to compare various designs for evaluating drug efficacy. The overall goal of the study was to optimize resource use by minimizing bias and maximizing precision (ie, minimizing uncertainty) of drug efficacy estimates, given a finite set of available resources. To make a fair comparison of the different survey designs, we constrained them to the same budget, meaning that designs that required more stool/urine samples or slides/urine aliquots to be examined per person could include fewer individuals. Our simulations use a Monte Carlo approach, which involves repeatedly simulating populations of individuals with “true” but unobserved baseline egg densities and examining the properties of the distribution of results obtained. To each simulated population, we applied each survey design in the following steps: (1) draw observed baseline egg counts for each individual from a count distribution; (2) determine the number of individuals included in the survey, given the budget and survey design (potentially selecting individuals based on observed baseline egg counts); (3) simulate the decrease in true egg density as a result of drug administration; (4) simulate observed post–drug administration egg counts from a count distribution; and (5) calculate the estimated ERR for the simulated survey based on the relative difference in sample mean egg count before and after drug administration.

These steps were repeated for a range of assumptions about the distribution of pre–drug administration infection levels (true egg densities), temporal variation in egg densities within individuals (in absence of drug administration), diagnostic variation in egg counts, and true drug efficacy. Parameter values reflecting these assumptions were chosen with hookworm in mind, but the overall concepts and results are readily generalizable to other STH species and SCH. Hence, when we refer to “stool samples” and “KK,” the reader may also interpret these as “urine samples” and “urine aliquot,” respectively.

### Survey Designs

We consider 7 main survey designs, the first 2 of which can be considered references for the comparison of alternative designs. The first design is “no selection” (NS), which involves testing each individual before and after drug administration. At baseline, each person is tested by single KK; exploratory analyses showed that testing >1 KK at baseline (and therefore being able to test fewer persons overall) did not increase precision of drug efficacy estimates. For the post–drug administration testing, we consider 3 variants of the NS design in which either a single stool sample is tested once (NS_1 × 1_) or twice (NS_1 × 2_) per person, or 2 samples are tested once each (NS_2 × 1_) per person. These designs are unbiased, have no specific aim to optimize resource use, and can be considered the simplest possible approach conceptually. The second survey design is “screen and select” (SS), which is the WHO-recommended design used in many studies as discussed in our introduction. Individuals are screened by means of a single KK with the aim to include only egg-positive individuals at follow-up. The single KK used for screening is also used in the analyses of drug efficacy. Because this design results in the inclusion of a greater proportion of egg-positive individuals in the ERR calculations, it produces more precise (ie, less uncertain) estimates of drug efficacy compared to NS designs. However, the SS design is also susceptible to bias due to regression toward the mean resulting from reuse of the screening sample as the pretreatment sample.

The remaining 3 designs all aim to avoid bias and yet optimize resource use by selecting egg-positive individuals for follow-up. The first is “screen, select, and retest” (SSR), which, like SS, involves selecting egg-positive individuals based on an initial screening. However, selected individuals are tested again before drug administration with an independent single KK based on a second stool sample. After drug administration, selected individuals are retested regardless of the result of their second predrug administration KK (some of which are expected to be negative). For the post–drug administration testing, we again consider 3 survey design variants that either test a single sample once (SSR_1 × 1_) or twice (SSR_ 1 × 2_) per person, or test 2 samples once each (SSR_ 2 × 1_) per person. Drug efficacy is estimated using only the second pre– and post–drug administration KK (ie, excluding the screening samples).

### Budgetary Constraints

To fairly compare the different survey designs, we constrain all designs by the same budget. We define the budget in terms of units representing the monetary cost of collecting a single stool sample from an individual and testing it with 1 KK [18]. We assume that collecting an additional stool sample per person doubles the cost, but that preparing a second KK based on the same stool sample increases the cost by only 0.621 units, based on previous costing and simulation studies [18, 19]. For the sake of simplicity, we assume that all tested subjects come from a single location, ignoring the additional cost of potentially having to visit more schools if more unique individuals are tested as part of a specific survey design. For illustrative purposes, we consider a total budget of 1200 units. See Table 1 for an example of how this budget would be optimally allocated under the different survey designs.

**Table 1.** Example of Budget Allocation for Seven Survey Designs to Estimate Drug Efficacy in a Setting With Expected Infection Prevalence of 10%

Design	Baseline Sampling Design		Follow-up Sampling Design		Budget Used
	*N*_0_	$S_{0}\times s_{0}$	*N*_1_	$S_{1}\times s_{1}$	
No selection (NS)					
Variant 1 (NS_1 × 1_): $N_{0}=B/2$	600	1 × 1	600	1 × 1	1200
Variant 2 (NS_2 × 1_): $N_{0}=B/2$	400	1 × 1	400	2 × 1	1200
Variant 3 (NS_1 × 2_): $N_{0}=B/\left(2+c\right)$	457	1 × 1	457	1 × 2	1197
Screen and select (SS): $N_{0}=B/\left(1+p\right)$	1091	1 × 1	109	1 × 1	1200
Screen, select, and retest (SSR)					
Variant 1 (SSR_1 × 1_): $N_{0}=B/\left(1+2p\right)\;$	1000 + 100	1 × 1	100	1 × 1	1200
Variant 2 (SSR_2 × 1_): $N_{0}=B/\left(1+3p\right)$	923 + 92	1 × 1	92	2 × 1	1199
Variant 3 (SSR_1 × 2_): N0 = B/ (1 + (2 + c)p)	950 + 95	1 × 1	95	1 × 2	1199

We assume a fixed budget of 1200 units, where 1 unit is equal to the monetary cost of collecting a single stool sample and performing a single Kato-Katz thick smear (KK) on it. Collecting an additional stool sample for a person and testing it with single KK is assumed to cost a full additional cost unit, whereas testing a second KK on the same sample is assumed to cost *KK* units [18, 19]. Baseline (*t=0*) and follow-up (*t=1*) sampling designs are defined in terms of *N*_*t*_ and $S_{t}\times s_{t}$, where *N*_*t*_ is the number of individuals, *S*_*t*_ is the number of fecal samples per individual, and *s*_*t*_ is the number of KK examined per sample. Formulae describe how the number of initially tested individuals (*N*_0_) is calculated, given the available budget (*B=1200*) and the expected fraction of egg-positive individuals (*P=.1*) as measured by an $S_{0}\times s_{0}=1\times 1$ design. A plus sign in the second column (*N*_0_) indicates that a subset of egg-positive individuals is tested a second time before drug administration, based on a new stool sample; the first stool sample is not used when estimating drug efficacy. The number of individuals tested at follow-up (*N*_1_) is always divided by the cost of follow-up testing per person. The total budget used is $\sum_{t=0}^{1}\left(N_{t}\times S_{t}\right)\left(1+c\times \left[s_{t}-1\right]\right)$. All results are rounded down to the nearest integer.

### Simulation Model

Data were simulated from a compound gamma-gamma-gamma-Poisson distribution, considering the 4 most relevant sources of variability to the overall distribution of egg counts:

1. Variability in mean egg intensity between individuals within a population due to variation in infection levels between individuals (assumed to be gamma-distributed with coefficient of variation cv_i_ = 1.5);
2. Day-to-day variability in mean egg intensity within an individual due to, for example, heterogeneous egg excretion over time (assumed to be gamma-distributed with cv_d_ = 0.75);
3. Variability in mean egg intensity between multiple KK based on the same nonhomogenized stool sample due to the aggregated distribution of eggs in feces (assumed to be gamma-distributed with cv_s_ = 0.25);
4. Variability in count observations due to random diagnostic variation (assumed to be Poisson-distributed).

The third and fourth level, together, are exactly equivalent to describing counts from repeated KK based on the same stool sample as following a negative binomial distribution. If a stool sample is homogenized before KK preparation, cv_s_ can be assumed to be closer to 0 such that egg counts from repeated slides based on the same stool sample are closer to Poisson-distributed [18]. Note that we follow Denwood et al [20] in the use of the coefficient of variation as a standardized measure of the variability of continuous distributions. The coefficient of variation can be related to the shape parameter κ of a gamma distribution by taking $\kappa =\frac{1}{cv^{2}}$ . Estimates of the coefficient of variation used here were arbitrarily chosen based on the authors’ experience of the typical host-parasite systems of interest.

Baseline egg counts in individual *i*, day *d*, and sample *s* were therefore simulated as:


$$\begin{array}{l} \begin{array}{ccc} \mu_{i} & \sim & \Gamma (k_{i}{}_{,\frac{\mu }{ki}}) \end{array}\\ \begin{array}{ccc} \mu_{id} & \sim & \Gamma (k_{d,}\frac{\mu_{i}}{k_{d}}) \end{array}\\ \begin{array}{ccc} \mu_{ids} & \sim & \Gamma (k_{s}\frac{\mu_{i}}{k_{s}}.w_{sample}) \end{array}\\ baseline\;count_{ids}\sim Pois(\mu_{ids}) \end{array}$$

Here, *µ* represents the average baseline fecal egg density (EPG) at the population level; *µ_i_* and *µ_id_* represent the expected EPG for an individual on any day and 1 particular day, respectively; *W_sample_* is the weight (or volume) of the biological sample under consideration; *µ_ids_* indicates the expected egg count for a single KK; κ is the shape parameter of a gamma (Γ) distribution; and *Pois* indicates the Poisson distribution. Higher values of κ indicate a lower coefficient of variation and therefore less overdispersion. We note that a compound gamma-Poisson distribution has the same distribution function as the negative binomial distribution. However, we also note that while the mean and variance of a compound gamma-gamma distribution are simple to calculate [20], the resulting distribution is not itself identical to a gamma distribution, so the overall distribution of egg counts we simulate is not exactly negative binomial.

Posttreatment egg counts were generated using a similar process, but using a mean that was scaled by a constant *r* reflecting the true anthelmintic efficacy:


$$\begin{array}{l} \begin{array}{ccc} V_{id} & \sim & \Gamma (k_{d}{}_{,\frac{\mu_{{}_{i}}.r}{k_{d}}}) \end{array}\\ \begin{array}{ccc} V_{ids} & \sim & \Gamma (k_{s,}\frac{V_{id}}{k_{s}}.W_{Sample}) \end{array}\\ follow-up\;count_{ids}\sim Pois(V_{ids}) \end{array}$$

Using the above process, we simulated 2 baseline (*μ_id_*) and 2 follow-up samples (*V_id_*) for a total of 1200 individuals (corresponding to the maximum budget), and for each sample we simulated 2 repeated KK. We chose the population average egg count (*µ*) to be 3.16, 5.45, 8.45, 12.0, or 23.5 EPG, such that given the chosen coefficients of variation, the apparent baseline prevalence of infection (ie, the prevalence of egg-positive individuals based on a single KK) was 10%, 15%, 20%, 25%, or 35%, respectively. Egg counts based on the first baseline sample were used as either baseline result (NS designs), for screening and selection of egg-positive individuals (SSR), or for both screening and as baseline result (SS). Egg counts based on the second baseline sample were only used in the SSR designs as baseline result. The individuals selected for each survey design were then chosen sequentially based on the budget available, and the group-based arithmetic mean ERR was calculated using the recommended procedure [4]. The ordering of individuals was kept consistent such that estimated ERRs were exactly comparable between survey designs for the same iteration. This process was repeated for a total of 5000 iterations for each combination of pretreatment prevalence of infection and true anthelmintic efficacy (true ERR ranging from 5% to 95% with 5%-point increments as well as 99%). The median bias of the estimator was calculated as the median difference between the observed ERR and true efficacy, and the standard deviation of the estimator was calculated as the standard deviation of the observed ERR.

All simulations and calculations were performed using a bespoke package for the statistical programming language R [21]. This package allows the same calculations to be made for any arbitrary set of parameter values, and is made freely available via http://ku-awdc.github.io/eggSim and https://www.fecrt.com/surveys/.

## RESULTS

**Figure 1.**
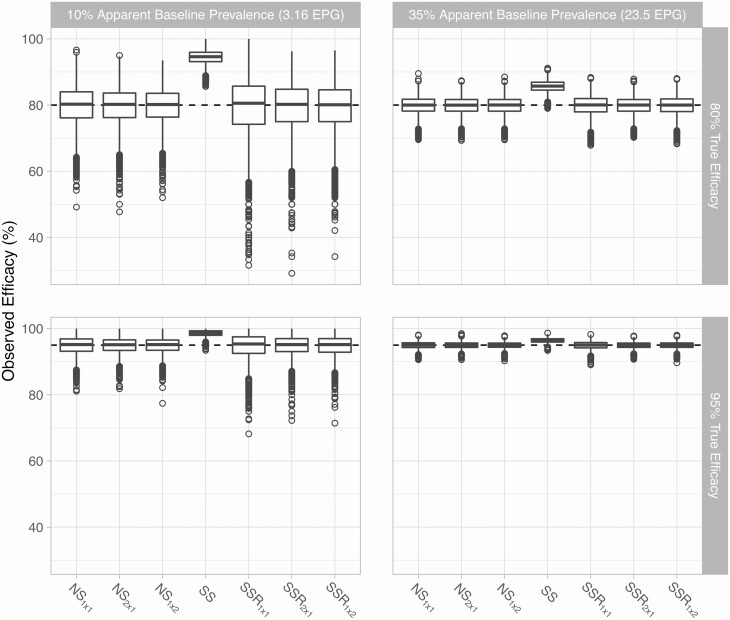
Estimated egg reduction rate vs the true value by sampling design and infection level. Boxplots show the median and interquartile range of estimated egg reduction rates from 5000 repeated simulations; whiskers cover the range of simulated values up to a distance of 1.5 times the interquartile range from the outer hinges of each box; open circles represent individual simulation results beyond the range of the whiskers. Simulations represent a setting with a pretreatment prevalence of egg positivity of about 10% (as measured by single Kato-Katz thick smear [KK]), true drug efficacy of 95% (horizontal dashed line), and a total budget equivalent to the cost of collecting and testing 1200 single KK. The “no screening” and “screen, select, and retest” designs each have 3 variants in which testing at follow-up is based on either a single KK (subscript 1 × 1), 2 slides based on 2 different fecal samples (2 × 1), or 2 KK based on the same stool sample (1 × 2). In the “screen and select” design, follow-up testing is based on a single KK. Abbreviations: EPG, eggs per gram of stool; NS, no screening; SS, screen and select; SSR, screen, select, and retest.

Using the same sample for both selection of egg-positive individuals and the baseline egg count (ie, the SS design) leads to an upward bias and higher precision (lower uncertainty) in the estimated ERR, in comparison to the unbiased naive designs without selection (NS) (Figure 1). If after selection of egg-positive individuals, a second baseline sample is collected and used for analysis and the first sample is excluded from analysis (SSR designs), the upward bias in ERR estimates is completely negated. However, this tends to come at the cost of lower precision compared to the NS design.

**Figure 2.**
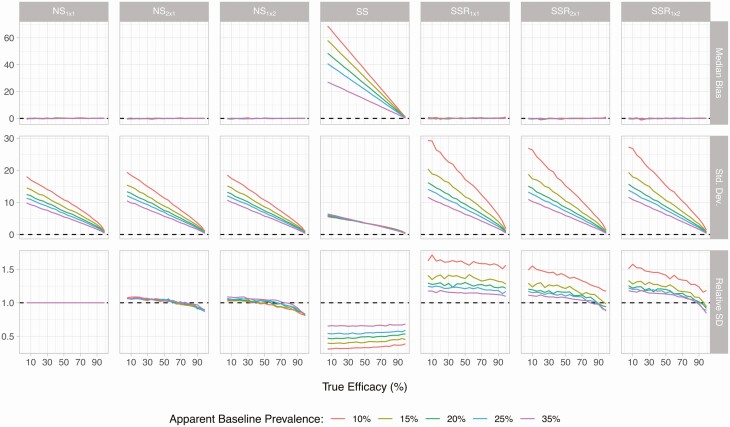
Bias and precision of estimated drug efficacy as a function of true drug efficacy and baseline prevalence by sampling design. Lines represent the median bias in egg reduction rate (ERR) estimates (top row), the standard deviation (SD) of ERR estimates (middle row), and the relative SD of estimates relative to the SD based on the NS_1 × 1_ design. Results are based on 5000 repeated simulations, assuming a total budget equivalent to the cost of collecting and testing 1200 single Kato-Katz thick smears (KK). The “no screening” and “screen, select, and retest” designs each have 3 variants in which testing at follow-up is based on either a single KK (subscript 1 × 1), 2 slides based on 2 different fecal samples (2 × 1), or 2 KK based on the same stool sample (1 × 2). In the “screen and select” design, follow-up testing is based on a single KK. Abbreviations: NS, no screening; SD, standard deviation; SS, screen and select; SSR, screen, select, and retest.

Among the 3 different variants of each the NS and SSR designs, ERR estimates were slightly more accurate for designs relying on >1 egg count per person at follow-up (Figure 1), despite the fact that fewer individuals could be tested within the budget constraints (Table 1). For the NS designs, precision was slightly higher when testing 2 KK based on the same sample (1 × 2), compared to testing 2 stool samples with single KK (2 × 1) at follow-up. This is most clearly visible in the first 3 panels in the bottom row of Figure 2, which show the standard deviation of the ERR estimator for each survey design, relative to that of the NS_1×1_ design. For the SSR designs, the variants employing >1 egg count at follow-up (1 × 2 and 2 × 1) performed very similarly in terms of standard deviation of the ERR estimator, although the 2 × 1 design produced an ERR distribution with a fatter left tail (Figure 1).

The upward bias in ERR estimates resulting from the SS design increased with lower true drug efficacy and lower pretreatment infection level (Figure 2). When the drug is almost completely ineffective, the SS strategy may yield estimates of drug efficacy as high as 60% in low-endemicity settings. This overestimation is not present in the NS design and is completely eliminated by SSR designs, despite the fact that egg-positive individuals are selected for follow-up.

Given the chosen coefficients of variation, the precision of ERR estimates based on NS designs employing multiple follow-up egg counts per person was consistently better than the precision of ERRs based on SSR designs. The NS_2×1_ and NS_1×2_ designs even outperformed the basic NS_1×1_ design in terms of precision if the true drug efficacy was ≥60%, particularly in low-endemicity settings. In contrast, the SSR_2×1_ and SSR_1×2_ designs only outperformed the NS_1×1_ design in more highly endemic settings (35% baseline prevalence, purple lines) and in case of high drug efficacy (≥90%). For the SSR design with the highest precision and slimmest left tail (ie, 1 × 2) and the NS design employing the same number of follow-up KK, we explored whether precision could be improved by somehow reducing the additional cost of a second KK, allowing inclusion of more individuals. Although the precision of results from the NS_1×2_ design improved with decreasing cost of the second KK, the precision of results from the SSR_1×2_ design hardly changed (Supplementary Figure 1) because the number of individuals tested at follow-up only increased marginally with lower cost of the second KK. For instance, at a 10% apparent baseline prevalence, the number of tested individuals increased only from 95 to 99 when the cost of the additional KK was 0.1 instead of 0.612, which is due to the majority of the budget being spent on screening either way.

## Discussion

Selection of individuals based on baseline egg counts and using those same egg counts in analyses of drug efficacy leads to inflated ERR estimates due to regression to the mean. This bias is particularly prominent in settings with low infection levels or when drug efficacy is low. Systemically overestimating drug efficacy is a potentially extremely dangerous outcome and we therefore strongly recommend that this study design not be used when the estimated ERR needs to be compared to other studies (either from the same or other areas) or a standard reference value. This bias can be eliminated by retesting initially selected individuals based on a new stool sample and excluding the first sample used for selection from the analysis, although this comes at the cost of reduced precision of the estimator. Precision of estimates can be further improved by increasing the number of posttreatment tests per person, despite the fact that fewer individuals can be tested within the same budget. Although the best alternative sampling design (SSR_1×2_) can achieve a level of precision close to a design without any selection (NS_1×1_), the resulting distribution of ERR estimates has fatter tails. As such, a simple design without selection and >1 follow-up egg count per person (NS_2×1_ or NS_1×2_) is theoretically the best to produce an unbiased estimate of the highest possible precision for a given budget. A potential benefit of NS designs is that loss to follow-up may be lower than for SSR designs as the NS design takes fewer days to execute. On the other hand, an SSR design may ensure that at least 1 stool sample is collected per individual. Ultimately, the choice of NS vs SSR will depend on the variance structure (ie, the coefficients of variation), which will depend on the parasite species and may well vary between geographical settings. Regardless, the NS and SSR designs in themselves are free from bias and therefore produce results that are more readily comparable across different settings and to the WHO standards for sufficient drug efficacy.

The ability of SSR design to negate upward bias in drug efficacy estimates due to regression toward the mean depends on how closely correlated repeated measurements are within individuals over time. With higher random variation over time (ie, lower correlation between subsequent days), the effect of regression to the mean based on a single sample will be stronger, but less time will be needed between screening and baseline samples to fully negate the bias. Given that daily variation in egg counts is high [18], we expect that for STH and SCH, retesting as soon as the next day is sufficient. More in-depth simulations for specific helminth species, informed by field data, are needed to make more definite statements about the optimal design for individual species. Such field data would ideally include repeated egg counts over time from the same individuals (without interventions such as treatment), based on multiple stool samples and multiple KK per sample. For STH and SCH, several published [22] and unpublished historical data sets already exist and should be further analyzed to establish realistic coefficients of variation and inform survey design in more detail. It is important to note that these coefficients of variation may even have to be allowed to vary between different endemic settings, as it was recently shown that the level of overdispersion of STH populations varies with baseline endemicity [23].

Although this study provides a strong recommendation against the SS design, we are reluctant to express a definitive preference for the NS and SSR based on statistical properties alone as other more practical factors may also affect the choice of survey design, such as logistical considerations (which we did not consider in detail in our budget allocation scheme). Also, secondary study objectives such as requiring a minimal number of eggs or egg-positive individuals for genotyping to identify resistance-conferring polymorphisms may be a reason to choose an SSR design as it will include more egg-positive individuals than an NS design based on the same budget. We also note that an SS design may be appropriate as long the data are analyzed using a statistical method that is able to account for missing-not-at-random data, such as the model used for “screened data” as presented by Geurden et al [24] for veterinary applications. However, it remains to be evaluated whether such an approach is feasible in the human context as it requires information on temporal variation based on a sufficient number of repeated egg counts per individual.

The issue of biased efficacy estimates due to selection is not as important for randomized clinical trials as for epidemiological surveys, because the effect of regression to the mean should be equivalent between trial arms. However, it may be relevant for cluster-randomized trials as the level of overdispersion (coefficients of variation) will likely vary between endemic settings and thus between clusters [23]. Theoretically, the bias would be more severe in clusters where temporal variation is higher relative to the mean; however, the cited study does not and cannot provide evidence of whether higher levels of overdispersion are (partially) driven by increased levels of temporal variation (relative to the mean) or not. We speculate that factors unrelated to parasite biology but that do vary between settings and cultures, such as laboratory procedures (eg, the extent to which stool is homogenized before preparing thick smears) and notably diet, may influence temporal variation in egg counts due to inhomogeneous distribution of eggs in stools. If this is the case, international cluster-randomized trials may benefit from using NS or SSR designs instead of an SS design to avoid differences in bias across clusters. Also, selection of egg-positive individuals is common practice in randomized clinical trials, which means that the actual estimates of drug efficacy from such trials should be interpreted with care and cannot be compared with estimates from field settings.

Regression toward the mean turned up as a unexpected finding in a recent analysis [18] of hookworm data from Tamil Nadu [25] where eggs were counted with McMaster on 3 different days after initial selection of egg-positive individuals based on a flotation test using the first of the 3 stool samples. In this study, mean egg counts were highest when based on the McMaster based on the first stool sample only, and were lower when egg counts were averaged over the 3 samples, indicating that egg counts had on average decreased over time in the selected individuals (without treatment). As a result, the conclusion of whether or not the prevalence of moderate-to-heavy intensity infection was above or under the WHO target for STH morbidity control (which was 1% at that time, currently 2%), depended on whether egg counts were based on the first sample only or all 3 samples. This finding highlights that extreme care is required when interpreting epidemiological survey results that involve selection of egg-positive individuals.

We note 3 major assumptions inherent to our method of calculation. First, we assume that the gamma distribution provides a reasonable approximation to the true distribution of infection/egg intensity at each variability partition, and that the diagnostic variability of counting eggs is entirely described by a Poisson distribution. Given the almost ubiquitous use of a negative binomial distribution to describe egg counts within the literature, we feel that this assumption is consistent with other studies. Second, we assume that all individuals have a nonzero pretreatment infection intensity, and that this is scaled by a fixed amount for all individuals following treatment. This assumption would not hold in situations where the pretreatment data is better described by a zero-inflated distribution, or where anthelmintic efficacy could be expected to vary between individuals either randomly or in a way that is correlated (negatively or positively) to the individual’s baseline infection intensity. The software we provide provides the facility to relax some of these assumptions, but further work is required to explore the implication of these effects on the overall bias and variance of the ERR estimator. Finally, we assume that the arithmetic mean ERR is the most appropriate method of summarizing these data. Our software also provides the facility to specify the desired ERR in terms of the geometric mean, and to calculate the observed ERR according to a geometric mean (with arbitrary fixed constant to avoid the inclusion of zeros). A more exhaustive comparison of these estimators, along with a broader discussion of this complex topic, is reserved for future work. As part of this future work, we will also consider more realistic budget allocation based on highly detailed and setting-specific costing data (eg, cost of personnel, materials, community sensitization, vehicles, and petrol).

Apart from STH and SCH, our findings and the framework that we developed are highly relevant to the filariases as well. Classical parasitological techniques such as counting of microfilariae in skin biopsies (onchocerciasis) or blood samples (lymphatic filariasis) still lie at the basis of many clinical trials and may also be employed in future drug efficacy surveys. As microfilarial counts will vary over time within individuals (without treatment), if only because of diagnostic Poisson variation (although higher levels of variation should be expected), the SS design comes with the same risk of bias as for STH and SCH species.

In conclusion, surveillance for timely detection of reduced drug efficacy should not be based on the conventional designs involving selection of individuals who are egg-positive before treatment. We illustrate and provide alternative and yet simple survey designs based on retesting of egg-positive individuals, which avoids biased drug efficacy estimates while also optimizing the use of available resources.

## Supplementary Data

Supplementary materials are available at *Clinical Infectious Diseases* online. Consisting of data provided by the authors to benefit the reader, the posted materials are not copyedited and are the sole responsibility of the authors, so questions or comments should be addressed to the corresponding author.

ciab196_suppl_Supplementary-Figure

## Notes

***Acknowledgments.*** The authors thank Johnny Vlaminck and Piero Olliaro for stimulating discussions during the Starworms meeting held on 24 January 2020 in Ghent, Belgium.

***Disclaimer.*** The views, opinions, assumptions, or any other information set out in this article are solely those of the authors and should not be attributed to the funders or any other person connected with the funders.

***Supplement sponsorship.*** This article appears as part of the supplement “Sustainable control of neglected tropical diseases – surveillance and diagnostics”, sponsored by the NTD Modelling Consortium.

***Author contributions.*** L. E. C. and M. J. D. conceived the study, designed the experiments, ran simulations, and interpreted results. M. J. D. coded the R package *eggSim* that was used for the analysis; in parallel, L. E. C. developed separate simulation code to test and verify *eggSim* predictions. L. E. C. drafted the manuscript. All authors critically revised the manuscript for intellectual content, and read and approved of the final manuscript.

***Financial support.*** L. E. C. and M. W. gratefully acknowledge funding of the Neglected Tropical Diseases Modelling Consortium by the Bill & Melinda Gates Foundation (grant number OPP1184344). L. E. C. further acknowledges funding from the Dutch Research Council (NWO; grant number 016.Veni.178.023). B. L. gratefully acknowledges support of the Starworms project through a grant from the Bill & Melinda Gates Foundation (grant number OPP1120972; B. L., principal investigator). The discussion meeting held on 24 January 2020 in Ghent, Belgium, that inspired the design of this study was partially funded through an FWO International Coordination Action (award number G0F4320N). M. W. reports grants from Research England and the Wellcome Trust, during the conduct of the study.

***Potential conflicts of interest.*** M. J. D. is involved in agricultural research including use of anthelmintics in livestock species. All other authors report no potential conflicts of interest.
