# Supplementary material for: Survey Design to Monitor Drug Efficacy for the Control of Soil-Transmitted Helminthiasis and Schistosomiasis
Source: Clin Infect Dis. 2021 Jun 14;72(Suppl 3):S195–202. doi: 10.1093/cid/ciab196 (PMC8201552; doi:10.1093/cid/ciab196)

SUPPLEMENTARY MATERIAL TO:

Survey design to monitor drug efficacy for the control of soil-transmitted helminthiasis and schistosomiasis

Luc E. Coffeng,[a#] Bruno Levecke,[b] Jan Hattendorf,[c,d] Martin Walker,[e] Matthew J. Denwood[f#]

[a] Department of Public Health, Erasmus MC, University Medical Center Rotterdam, Rotterdam, The Netherlands

[b] Department of Virology, Parasitology and Immunology, Ghent University, Merelbeke, Belgium.

[c] Swiss Tropical and Public Health Institute, Basel, Switzerland

[d] University of Basel, Basel, Switzerland

[e] Department of Pathobiology and Population Sciences and London Centre for Neglected Tropical Disease Research, Royal Veterinary College, Hatfield, UK

[f] Department of Veterinary and Animal Sciences, University of Copenhagen, Denmark

[#] Corresponding author: Luc E. Coffeng, Department of Public Health, Erasmus MC, University Medical Center Rotterdam, P.O. box 2040, 3000 CA Rotterdam, The Netherlands; l.coffeng@erasmusmc.nl. Alternate correspondence: md@sund.ku.dk.

Supplementary Figure 1. Precision of estimated drug efficacy as a function of true drug efficacy and baseline prevalence, assuming that the costs of a second Kato-Katz thick smear (KK) is either 0.1 units, 0.621 units (as in main analysis), or 1 unit. Lines represent the relative standard deviation of drug efficacy estimates relative to the standard deviation of estimates based on the NS_{1x1} design. Results are based on 5,000 repeated simulations, assuming a total budget equivalent to the cost of collecting and testing 1,200 single KK. SS = Screen and Select; SSR = Screen, Select, and Retest; each employ two follow-up KK based on the same stool sample (1x2).

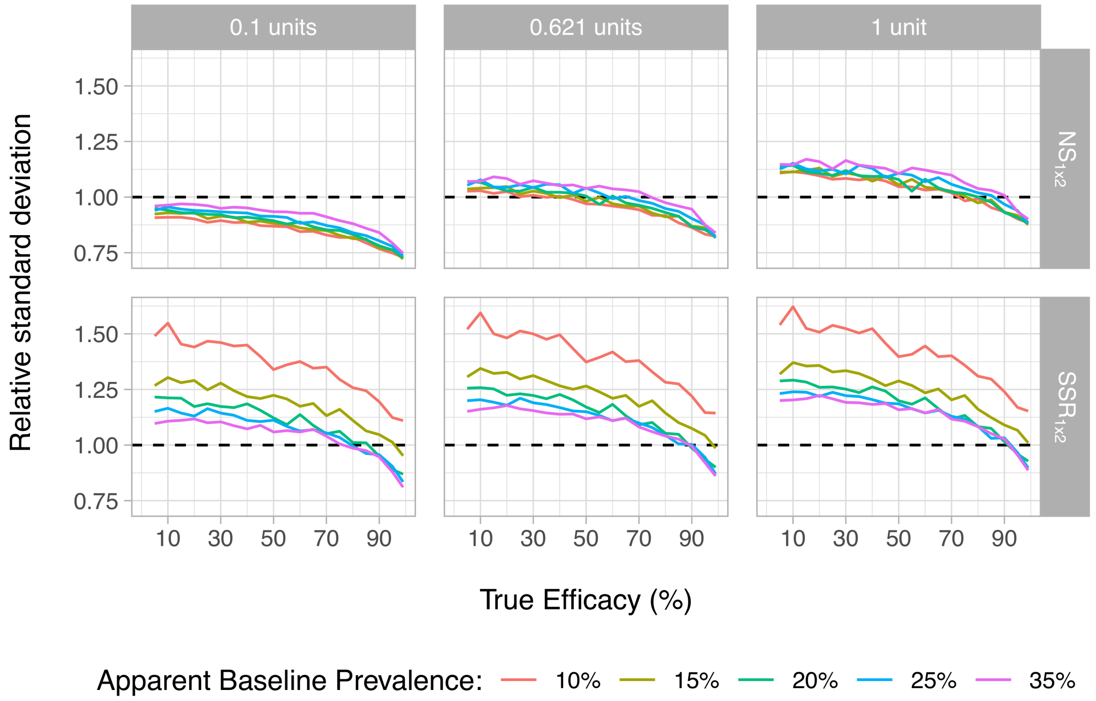

Supplement: ciab196_suppl_Supplementary-Figure [file ciab196_suppl_supplementary-figure.pdf]
